# Genome-Wide Characterization and Expression Profiles of the Superoxide Dismutase Gene Family in* Gossypium*

**DOI:** 10.1155/2016/8740901

**Published:** 2016-08-31

**Authors:** Jingbo Zhang, Bo Li, Yang Yang, Wenran Hu, Fangyuan Chen, Lixia Xie, Ling Fan

**Affiliations:** ^1^Institute of Nuclear and Biological Technologies, Xinjiang Academy of Agricultural Sciences, 403 Nanchang Road, Urumqi 830091, China; ^2^School of Life Sciences, Xinjiang Normal University, 102 Xinyi Road, Urumqi 830054, China

## Abstract

Superoxide dismutase (SOD) as a group of significant and ubiquitous enzymes plays a critical function in plant growth and development. Previously this gene family has been investigated in* Arabidopsis* and rice; it has not yet been characterized in cotton. In our study, it was the first time for us to perform a genome-wide analysis of SOD gene family in cotton. Our results showed that 10 genes of SOD gene family were identified in* Gossypium arboreum* and* Gossypium raimondii*, including 6 Cu-Zn-SODs, 2 Fe-SODs, and 2 Mn-SODs. The chromosomal distribution analysis revealed that SOD genes are distributed across 7 chromosomes in* Gossypium arboreum* and 8 chromosomes in* Gossypium raimondii*. Segmental duplication is predominant duplication event and major contributor for expansion of SOD gene family. Gene structure and protein structure analysis showed that SOD genes have conserved exon/intron arrangement and motif composition. Microarray-based expression analysis revealed that SOD genes have important function in abiotic stress. Moreover, the tissue-specific expression profile reveals the functional divergence of SOD genes in different organs development of cotton. Taken together, this study has imparted new insights into the putative functions of SOD gene family in cotton. Findings of the present investigation could help in understanding the role of SOD gene family in various aspects of the life cycle of cotton.

## 1. Introduction

Cotton (*Gossypium* spp.) is not only an important economic crop, but also an excellent model plant for the study on genome polyploidization, epidermal cell fate determination, cell elongation, and cell wall development [1–4]. As the most important fiber and oil crop, cotton is widely cultivated in more than 100 countries. Cotton fiber and seeds have become the indispensable source for textile industry and catering industry around the world [5].

*Gossypium hirsutum* as the major cotton species has contributed 90% yield of cotton in worldwide [6]. In evolution history,* Gossypium hirsutum* is the allotetraploid species which is originated from the A-genome species (*Gossypium arboreum*,* G. arboreum*) as the maternal parent and D-genome species (*Gossypium raimondii*,* G. raimondii*) as the male parent [7]. The* G. arboreum* can produce fiber. Nevertheless,* G. raimondii* do not generate fiber. Therefore, it is generally considered that the character of A-genome species maybe determined the property of* G. hirsutum* fiber; 1-2 million years ago, the diverged* G. arboreum and G. raimondii* were reunited, which resulted in the formation of the* G. hirsutum* [8].

Reactive oxygen species (ROS) are a series of byproducts which is produced by several essential aerobic reactions during metabolic processes in the chloroplasts, mitochondria, and peroxisomes [9]. Plants often face the challenge of serious biotic and abiotic stresses, which would also cause the accumulation of ROS. The excessive accumulation of ROS can result in oxidative damage or cell damage and will seriously influence growth and development of plant [10, 11]. On the other hand, ROS also can be used as signaling molecules in different organisms to coordinate an astonishing range of diverse plant processes, involved in regulation of plant growth and cell programmed death [12]. Generally, ROS keeps at physiologically optimal levels under normal conditions by an efficient ROS-scavenging system. The major ROS-scavenging enzymes are composed of superoxide dismutase (SOD), ascorbate peroxidase (APX), catalase (CAT), glutathione peroxidase (GPX), and peroxiredoxin (PrxR) [13]. These enzymes provide cells with highly efficient machinery for maintaining the dynamic balance of ROS concentration in plants [14].

Superoxide dismutase (SOD), the first enzyme involved in ROS-scavenging system, functions as the dismutase to catalyze superoxide (O_2_^−^) into molecular oxygen (O_2_) and H_2_O_2_ [15]. Plant SOD can be categorized into three groups according to the types of binding metal ions, including copper-zinc SOD (Cu/Zn-SOD), iron SOD (Fe-SOD), and manganese SOD (Mn-SOD). These SODs were distributed in different compartment of cell and play a critical role in response to oxidized stress [16]. Usually, most Fe-SODs and Cu/Zn-SOD are located in chloroplasts. Moreover, Cu-Zn-SOD was observed to be the most abundant during SODs. Interestingly, in chloroplast and cytosol, Cu/Zn-SODs are differentiated based on their numbers and positions of metal ion binding [17]. As for Mn-SOD, it is the SOD which is often found in peroxisomes and mitochondria [18]. Plant genomes were often expected to contain at least one copy of Mn-SODs in terms of their important roles in scavenging ROS in mitochondria [19].

In cotton, previous study demonstrated that overexpression of SODs increased the tolerance to salt stress and oxidized stress induced by methyl viologen, which indicated that SODs increased the cotton resistance to abiotic stress [20]. In addition, Cu-Zn-SOD was found in developing cotton fibers and transiently increasing the abundance at the transition period between cell elongation and secondary cell wall synthesis [21]. Another report implied that SOD might be involved in the differentiation of secondary walls in cotton fibers, because SOD catalyzed superoxide (O_2_^−^) to produce hydrogen peroxide which was a remarkable signal for the differentiation of secondary walls in cotton fibers [22]. These results indicated that the SOD gene family play a critical role in cotton fiber development.

Since releasing amount of publicly available data and even whole genome sequences in some plants, genome-wide analysis of the SOD gene family has been feasible. Previous report in* Arabidopsis* identified a total of 7 candidate SOD genes by genome-wide analysis, including 1 Mn-SOD (*MSD1*), 3 Fe-SODs (*FSD1*,* FSD2*, and* FSD3*), and 3 Cu-Zn-SODs (*CSD1*,* CSD2*, and* CSD3*) [23]. Furthermore, 8 SOD genes were reported in* Sorghum* and rice genome, respectively [24, 25]. However, SOD family members in cotton have not been comprehensively identified and characterized.

To gain insights into the structural and functional attributes of SOD family in cotton, present study investigated genome-wide characterization and expression profiles of superoxide dismutase gene family for the first time in* G. arboretum* and* Gossypium raimondii*. Our results showed that a total of 10 nonredundant SOD coding genes were identified in the genome of* G. arboretum* and* G. raimondii,* respectively. And then, they were subsequently subjected to a comprehensive analysis, including phylogenetic relationship, chromos distribution, gene duplication status, gene structure arrangement, conserved motif composition, expression pattern under different abiotic stresses, and expression profiles of different organs in cotton. In a word, our genome-wide analysis of SOD gene family will contribute to future studies on the functional characterization of SOD genes in cotton as well as comprehensive analysis of the SOD gene family in other species.

## 2. Materials and Methods

### 2.1. Plant Materials

Upland cotton (*G. hirsutum*) cultivar CRI 35 was cultivated in a normal agronomic field from May to September under standard condition in Beijing. The seeds were kindly provided by the Cotton Research Institute, Chinese Academy of Agricultural Sciences. Flowers were tagged on the day of anthesis. Cotton bolls were harvested at 0, 5, 10, 15, 20, 25, and 30 dpa, respectively. Ovules were excised from the bolls, and fibers were scraped from the ovules. The other tissues including root, stem, leaf, hypocotyls, petal, and anther were also collected. All collected materials were immediately frozen in liquid nitrogen and then stored at −80°C until RNA extraction.

### 2.2. Sequence Retrieval of Cotton SOD Genes

To identify SOD genes in* G. arboretum* and* G. raimondii*, all published SOD gene sequences of* Arabidopsis* and rice were employed to perform a homologous blast in the* G. arboretum* (http://cgp.genomics.org.cn/page/species/index.jsp) genome and* Gossypium raimondii* (http://www.phytozome.net/cotton) genome database as previously described [26]. Furthermore, the conserved SOD domains of predicted protein sequence were evaluated using InterProScan program and Pfam tools (http://www.sanger.ac.uk/software/pfam) according to reported method [27]. Additionally, the physicochemical characteristics of cotton SOD proteins were predicted by using the ProtParam tool (http://www.expasy.org/tools/protparam.html) as previously described [28]. The parameters of cotton SOD proteins involving the amino acid number, molecular weights (MWs), and theoretical isoelectric points (pIs) were calculated. Subsequently, subcellular localization of SODs was predicted by using WoLF PSORT (http://psort.hgc.jp/) and TargetP 1.1 (http://www.cbs.dtu.dk/services/TargetP/) tools as previously described [29].

### 2.3. Sequence Alignments and Phylogenetic Construction

Multiple sequence alignments were generated using Cluster X^19^ software for the amino acid sequences of 20 SOD proteins from* G. arboreum*,* G. raimondii*,* Arabidopsis*, rice,* Populus,* and* Sorghum* including 7* Arabidopsis* sequences, 8 rice sequences, 8 polar sequences, and 8* Sorghum* sequences. Based on the result of multiple sequence alignment, MEGA 6.0 software was employed to construct an enrooted phylogenetic tree using a neighbor-joining (NJ) tree method with 1000 replicates as previously described [30]. Then the enrooted phylogenetic tree was subjected to ITOL (http://itol.embl.de/) to form the interactive tree.

### 2.4. Gene Structure Analysis and Conserved Motif Identification

The genomic sequences of SOD genes and their coding sequences were retrieved from* G. arboretum* and* G. raimondii* genome database as previously described in Section 2.2 and were loaded into gene structure display server (http://gsds.cbi.pku.edu.cn/) to infer the exon/intron arrangement of SOD genes. All SOD protein sequences were submitted to SMART (http://smart.embl-heidelberg.de/) and Pfam (http://www.sanger.ac.uk/software/pfam) online tools to predict the conserved domain in SOD proteins. Then the conserved motifs in SOD proteins were detected by using Motif Elicitation (MEME) online program (http://meme.sdsc.edu/meme/intro.html) with the optimum width from 6 to 100 and maximum number of motifs 20. The functional annotation of the identified motifs was implemented with InterProScan tool (http://www.ebi.ac.uk/Tools/InterPro-Scan/).

### 2.5. Analysis of Chromosomal Location and Gene Duplication

In order to determine the chromosomal distribution of the SOD genes, physical locations of all cotton SOD genes on chromosomes were retrieved through the BLASTN searching for* G. arboretum* and* G. raimondii* genome database (http://cgp.genomics.org.cn/page/species/index.jsp) and showed in map using MapDraw 2.2 software [31].

In this study, gene duplication of SOD gene family was detected based on the criteria described as previous studies [32, 33]. In addition, to explore the mechanism of gene divergence, the software DnaSP 5.0 was applied to calculate the synonymous substitution rate (*Ks*) and nonsynonymous substitution rate (*Ka*). The data of duplication events were subsequently estimated according to the equation *T* = *Ks*/2*λ*. The approximate value for clock-like rate was 1.5 synonymous substitutions per 10^8^ years for diploid cotton [34].

### 2.6. Microarray-Based Expression Analysis and Correlation Calculation

To determine the expression patterns of SOD genes under abiotic stresses, whole genome microarray analysis was performed. Public cotton expression data were obtained from the Plant Expression Database (PLEXdb) and downloaded from the Gene Expression Omnibus (GEO) (accession number GSE50770, for abiotic stresses of abscisic acid (ABA), cold, salinity, and alkalinity; accession number GSE16467, for waterlog treatment; accession number GSE29810, for drought treatment), as previously described [35, 36].

The values of signal intensity were normalized and the average of biological replicate was used to generate log⁡2 expression value. Then log⁡2-transformed values were subjected to R software (15.2) for expression analysis. Limma package was used to analyze expression data and gplots package was used to generate heatmap represented log⁡2-transformed probe intensities.

### 2.7. RNA Isolation and Real Time PCR Analysis

To clarify the developmental and tissue-specific expression profiles of SOD genes in cotton, the quantitative real time PCR (qRT-PCR) was performed to detect the expression level of SOD genes at seven fiber development stages (0, 5, 10, 15, 20, 25, and 30 dpa.) and six organs (root, stem, leaf, hypocotyls, petal, and anther). The samples were grounded to powder using liquid nitrogen, and then total RNA of cotton tissues was extracted using Qiagen RNA Mini Kit according to the manufacturer's protocol. The total of 2 *μ*g of RNA was used as the template for the first-strand cDNA synthesis using an RNA PCR kit (TaKaRa, Otsu, Japan). The resulting cDNA products were diluted 1/5 and stored at −20°C for qRT-PCR analysis.

The specific primers for each* GaSOD* gene and reference gene* UBQ7* are showed in Table 1. Quantitative RT-PCR was performed with a MiniOpticon Real Time PCR System (Bio-Rad, CA, USA) according to the supplier's protocol; each reaction mixture contained 8 *μ*L of DNase/RNase-free water, 10 *μ*L of the 2x SYBR Green PCR master mix, 1 *μ*L of the diluted cDNA product whose concentration is 50 ng/*μ*L, and 1 *μ*L of the gene-specific primers whose concentration is 10 pM. The expression values of the SOD genes were normalized with an internal housekeeping gene* UBQ7* (gene accession number: DQ116441). Three biological replicates were conducted for each sample, and each biological replicate was technically repeated three times. The program applied for the thermal cycle was as follows: 95°C for 5 min, 40 cycles of amplification at 95°C for 5 s, 58°C for 30 s, and 70°C for 30 s. The relative expression levels were calculated using the comparative 2^−ΔΔCT^ method. Each PCR was run in triplicate in each assay. A heatmap of* GaSOD* genes expression profiles was produced with R program's pheatmap.

## 3. Results

### 3.1. Identification of SOD Gene Family in* G. arboretum* and* G. raimondii* Genomes

To identify SOD genes in* G. arboretum* and* G. raimondii*, all published SOD gene sequences of* Arabidopsis* and rice were employed to perform a homologous blast in the* G. arboretum* genome and* Gossypium raimondii* genome database. Our analysis has identified 10 SOD genes sequences in* G. arboretum* and* G. raimondii,* respectively. These 10 genes were divided into three subfamilies, including Cu-Zn-SOD (6 SOD genes), Fe-SOD (2 SOD genes), and Mn-SOD (2 SOD genes). Due to the lack of standard annotation, 10 SOD genes of* G. arboretum* were named as* GaCSD1* to* GaCSD6*,* GaFSD1* to* GaFSD2*, and* GaMSD1* to* GaMSD2* in terms of their locations on* G. arboretum* chromosomes (Table 2). Then, we designated SOD genes of* Gossypium raimondii* according to the highest sequence similarity with GaSODs.

Furthermore, conserved SOD domains were confirmed by using SMART and Pfam tools. The result showed that all of the putative SOD genes contained conserved SOD domain as the same of* Arabidopsis* and rice. Other characteristics of SOD genes, including isoelectric point (pI), molecular weight (Mw), chromosome location, and subcellular localizations were listed in Table 2. Interestingly, all Cu-Zn-SODs and Fe-SODs predicted in chloroplast and cytoplasm were classified into acidic amino acids, whereas all Mn-SODs were composed with alkaline amino acids and mostly located in mitochondria. The characters of amino acids and subcellular localizations reflected the function and divergence for SOD gene family of cotton.

### 3.2. Phylogenetic Analysis of SOD Proteins in* G. arboretum* and* G. raimondii*

**Figure 1:**
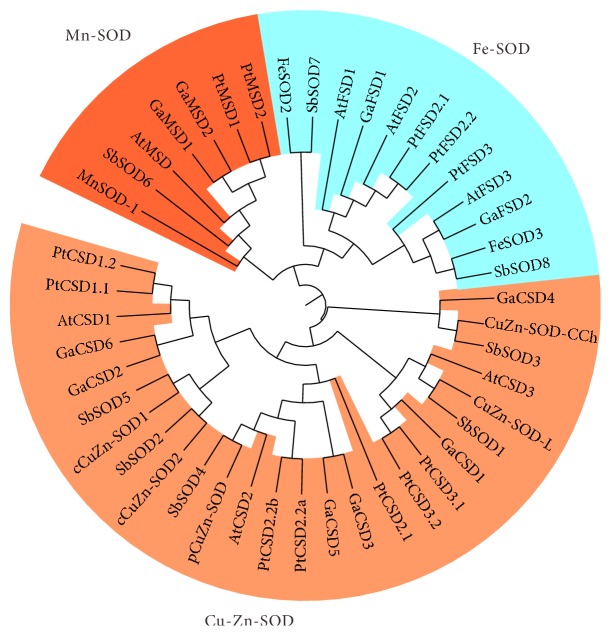
Phylogenetic relationships of SOD gene from* G. arboreum*,* G. raimondii*,* Arabidopsis*, rice,* populus, and Sorghum*. The three subfamilies are indicated with different colors. The CaSODs represent the 10 SOD genes in* G. arboreum*, GrSODs represent the 10 SOD gene in* G. raimondii*, the AtSODs represent the 7 SOD genes in* Arabidopsis*, PtSODs represent the 12 SOD genes in polar, Cu-Zn-SOD genes represent the 8 SOD gene in rice, and SbSODs represent the 8 SOD genes in* Sorghum*.

To obtain a better understanding of the evolutionary history and evolutionary relationships of SOD gene family in cotton, an enrooted phylogenetic tree was constructed with NJ method on the basis of multiple sequence alignment of 20 cotton SOD protein sequences including* Arabidopsis*, rice,* Populous* [35], and* Sorghum* (Figure 1). The relatively large number of sequences caused the reduction of bootstrap values for NJ tree. Therefore, three other methods including maximum likelihood, minimal evolution, and PhyML methods were used to reconstruct the phylogenetic tree. The phylogenetic tree reconstructed by the four methods was almost identical with each other.

Based on the phylogenetic tree, we could clearly observe that the SOD genes within the same subfamily were clustered together. However, the subgroup of cotton SOD genes were interspersed in most clades, showing that the SOD gene family was expanded before the divergence of the lineages. Moreover, we also could find that the dicot SODs (cotton,* populous,* and* Arabidopsis* SODs) have more closely phylogenetic relationship related to monocot SODs (rice and* Sorghum* SODs) in each clade with all plants.

### 3.3. Chromosomal Organization and Gene Duplication

**Figure 2:**
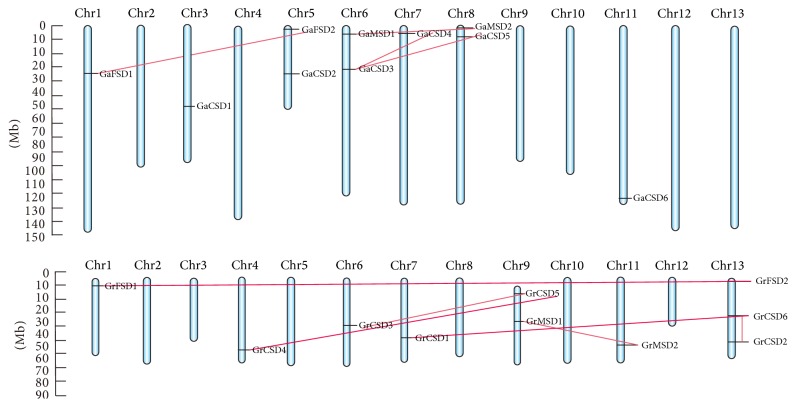
Chromosomal distribution and gene duplication of SOD genes in* G. arboreum* and* G. raimondii*. The scale is in megabases (Mb). The chromosome numbers are indicated at the top of each chromosome. The paralogous SOD genes are connected with a red line.

The chromosomal location of SOD genes was determined by retrieving from* G. arboretum* and* G. raimondii* genome, respectively; the gene loci showed that 10 GaSOD genes were distributed in 7 chromosomes (Figure 2). In summary, the number of SOD genes on each chromosome appeared to be unevenly. Chromosomes V, VI, and VII possessed two SOD genes each, accounting for 60% of the total GaSOD genes, while one SOD gene was distributed on chromosomes I, III, VII, and XI of* G. arboretum* genome.

These gene pairs are present in the same clade of the phylogenetic tree with high similarity. For example, the sequence of* GaMSD1* covers 94.5% sequence similarity with the sequence of* GaMSD2*. Among these paralogous gene pairs, 4 pairs in* G. arboretum* and 6 pairs in* Gossypium raimondii* were located on different chromosomes, suggesting that these genes might result from segmental duplication event during the evolution.

To illuminate the divergence after gene duplication, we calculated the synonymous substitution rate (*Ks*) and nonsynonymous substitution rate (*Ka*) of all duplicated genes in* G. arboretum* and* Gossypium raimondii* (see Table S2 in Supplementary Material available online at http://dx.doi.org/10.1155/2016/8740901). Nevertheless, there are 2 segmentally duplicated pairs that suffered positive selection with *Ka*/*Ks* ratios greater (show the value). Previous reports proposed that the doubling of gene content is a relax selection for individual genes in newly formed polyploidy, which also provides opportunities for novel gene evolution and expression patterns [29, 30]. We further calculated the approximate dates of duplication event. Our data indicated segmental duplications of* GaSOD* genes occurring between 12.43 Mya (million years ago) and 30.74 Mya; segmental duplications of* GrSOD* genes occurred between 9.13 Mya (million years ago) and 32.82 Mya with an average of 17.34 Mya (Table S1). The result indicated that the occurrence of duplication event was later than the division in cotton and* Arabidopsis* (83–86 mya) and was consistent with the time (20–40 mya) of large-scale genome duplication event that occurred in cotton.

### 3.4. Gene Structures and Conserved Motifs

**Figure 3:**
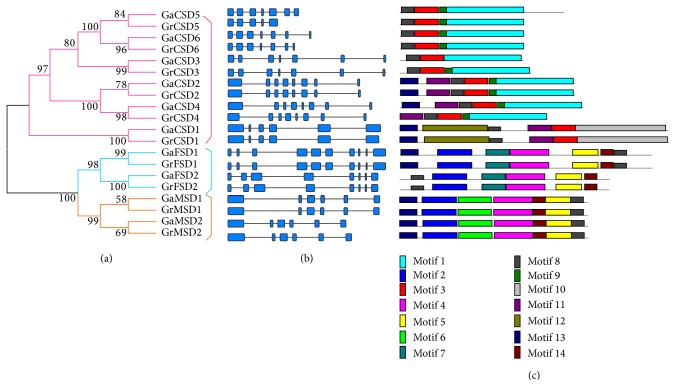
Phylogenetic analysis, gene structure, and conserved motifs of SOD gene family in cotton. (a) The phylogenetic tree of all SOD genes in* Gossypium arboreum*. (b) The exon/intron organization of SOD genes in* Gossypium arboreum*. The green boxes represent exons and black lines indicate introns. (c) The conserved protein motif of SOD gene family was identified. Each motif is indicated with a specific color.

It is well known that gene structural diversity is a possible mechanism for guiding the long-term evolution of multigene families. With the aim of gaining further insights into the evolutionary relationships among cotton SOD genes, we investigated the exon/intron structures of individual SOD genes by the alignment of cDNA sequences and corresponding genomic DNA sequences (Figure 3(b)). Additionally, an enrooted phylogenetic tree was constructed with SOD protein sequences to determine if the exon/intron arrangement of SOD genes is consistent with the phylogenetic relationship (Figure 3(a)). As expected, the SOD members in the same clade of phylogenetic tree demonstrated a very similar exon/intron distribution pattern. For example, the* GaMSD1* and* GrMSD1* had the same numbers of exon/intron and similar length.

To explore the motif compositions in SOD genes, motif structures were detected in SOD proteins analyzed by MEME motif search tool. A total of 14 motifs for SOD proteins were identified (Figure 3(c)). The same subfamily was observed with common motifs. In addition, the homologous genes of SOD gene family in G. arboretum and* G. raimondii* had shown high similarity in the composition of motifs. Owing to the unclear function of motifs for cotton* SOD* proteins, ScanProsite tool was employed to annotate the function of identified 14 motifs.

It was reported that the SOD gene family usually contain highly conserved domain involved in metal binding [37]. In order to clarify the structure of SOD protein, three groups of SOD protein sequences of cotton,* Arabidopsis,* and rice were subjected to SMART and Pfam (http://pfam.sanger.ac.uk/) tools. Our data indicated that all Cu-Zn-SODs hold a copper-zinc domain (Pfam: 00080). Particularly, the heavy-metal-associated domain (Pfam: 00403) was also found in all three groups of Cu-Zn-SODs. Additionally, all Fe-SODs and Mn-SODs possessed the iron/manganese superoxide dismutase alpha-hairpin domain (Pfam: 00081) and iron/manganese superoxide dismutase C-terminal domain (Pfam: 02777). However, there is worth noting that two Fe-SODs had two iron/manganese superoxide dismutases. As shown in Figure S1, the similar domain structure of SOD was exhibited in the same subfamily and different species. The result supported that the domain structure for SOD gene family was highly conserved in different species.

### 3.5. Abiotic Stress Inducible Expression of SOD Genes in* G. arboretum* and* G. raimondii*

**Figure 4:**
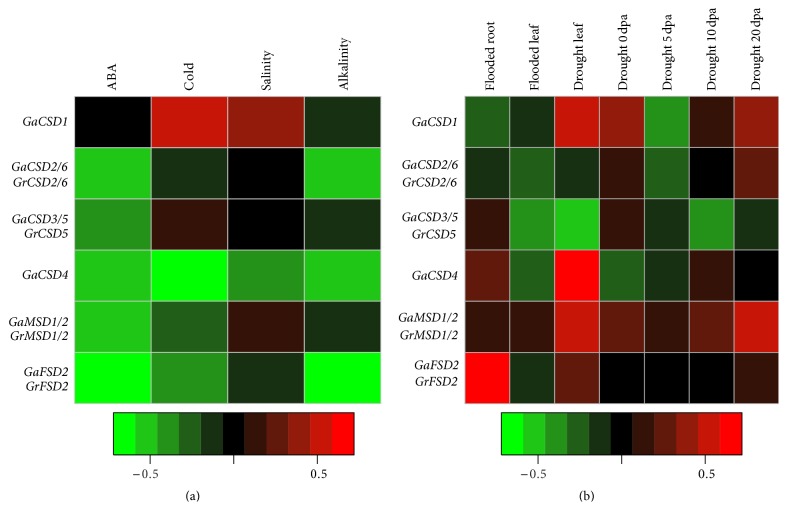
Expression profiles of cotton SOD genes under abiotic stress. (a) Expression patterns under cold, salinity, ABA, and alkalinity stresses (GSE50770) in cotton seedlings with different abiotic stresses treatment were selected at 14-day after germination. (b) Expression profiles in* G. hirsutum* roots and leaves under waterlog stress (GSE16467) and microarray expression data for leaf and fiber development stages (0, 5, 10, and 20 dpa) under drought stress (GSE29567 and GSE29810).

To determine the potential function of SOD genes in cotton development, microarray analysis was performed to detect the expression pattern under abiotic stresses involving abscisic acid (ABA), cold, salinity, and alkalinity. The result of microarray revealed that total of 11 SOD genes increased significantly under cold condition and high concentration of salinity (Figure 4(a)), such as* GaCSD2*,* GrCSD2*,* GaCSD6*,* GrCSD6*,* GaCSD3*,* GaCSD5*,* GrCSD5*,* GaMSD1*,* GaMSD2*,* GrMSD1,* and* GrMSD2*. Our data is consistent with previous study that SOD activities were elevated after cold stress and salt stress [38, 39] (supplement with the results of ABA and alkalinity treatment).

**Figure 5:**
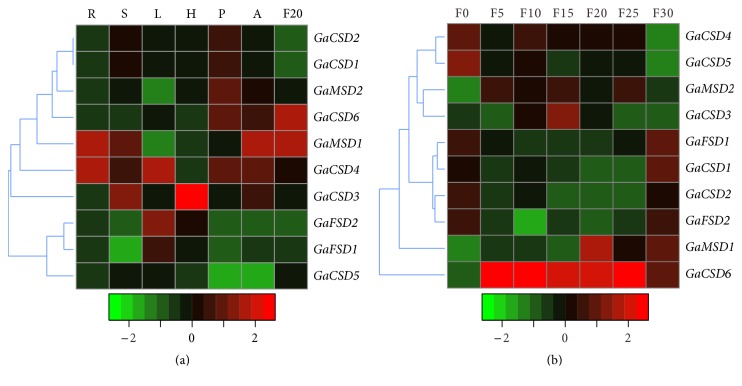
Expression patterns of GaSOD genes. (a) Quantitative RT-PCR of GaSOD genes in root (R), stem (S), leaf (L), hypocotyl (H), petal (P), anther (A), and fiber at 20 DPA (F20) of cotton plants. (b) Quantitative RT-PCR of GaSOD genes in cotton fibers at different developmental stages. F0, ovules from 0 DPA; F5 to F30, fibers from 5 to 30 DPA. Results were normalized using cotton UBQ7 gene expression as the internal control.

Furthermore, the expression patterns of SOD genes in cotton root and leaf were analyzed at fiber development stages (0, 5, 10, 15, 20, and 25 dpa) under waterlog and drought stresses. As shown in Figure 4(b), the waterlog and drought stresses induced most of SOD genes expression. Two SOD genes of* GaFSD2* and* GrFSD2* showed a significant improvement in flooded root. Five genes (GaCSD1, GaCSD4, GrCSD4, GaMSD1, and GrMSD2) elevated the expression level in drought leaf.* GaMSD1*,* GrMSD2,* and* GrCSD2* exhibited the higher values in each fiber development stage under drought stresses compared with normal conditions. These results showed the expression level of SOD genes induced by abiotic stresses in agreement with the reports in tobacco and liquorice [40–42] (Figure 5(b)).

### 3.6. Expression Profiles of SOD Genes in* G. arboretum* and* G. raimondii*

To clarify the developmental and tissue-specific expression profiles of SOD genes in cotton, the quantitative real time PCR (qRT-PCR) was performed to detect the expression level of SOD genes at seven fiber development stages (0, 5, 10, 15, 20, 25, and 30 dpa.) and six organs (root, stem, leaf, hypocotyls, petal, and anther).

Previous study had reported that photosynthetic electron transport chain (PET) operates in an aerobic environment [43]. As shown in Figure 5(a), SOD genes were differentially expressed in tested tissues, while we found many SOD genes were highly expressed in leaf. Our finding implied that SOD as a major ROS-scavenging enzyme may be involved in the photosynthesis of plant. In addition, we also detected the expression levels of other SOD genes were high in other tissues; for example,* GaCSD4* and* GaMSD1* have highly expression level in root and* GaCSD3* have highly expression level in hypocotyls. The result indicated that the SOD genes play important roles in the different tissues development.

The development process of cotton fiber mainly involved four overlapping stages, including fiber initiation, cell elongation, secondary wall deposition, and maturation [44, 45]. We further investigated the expression regulation of SOD genes at different fiber development stages. The expression levels of* GaCSD1*,* GaCSD2*,* GaCSD4*,* GaCSD5*,* GaCSD7,* and* GaCSD8* were high in 0 dpa ovule. We can find the expression level of* GaMSD1* was high during 5 dpa–10 dpa, indicating it participates in the cell elongation of fiber. The expression levels of* GaCSD1*,* GaFSD1*,* GaFSD2,* and* GaFSD2* were higher at 30 dpa fiber suggesting they are involved in secondary wall deposition and maturations of fiber. These results implied that SOD genes have important functions in development process of cotton fiber.

### 3.7. Discussion

SOD plays crucial roles in multiple process of plant growth and against environment stresses. However, only a tiny fraction of SOD genes have been identified in plants. Genome-wide analysis is an important approach for elucidating the biological roles of the SOD gene family members in given plant species. The previous availability of genome sequences has enabled comprehensive analysis of this gene family in* Arabidopsis*, rice,* Populous,* and* Sorghum*. However, no SOD gene family have been characterized in cotton. This study has conducted a genome-wide analysis of the SOD gene family in* G. arboretum* and* G. raimondii*.

The gene duplication is an important way for the expansion of gene families [46]. Recent studies have shown that* G. arboretum* and* G. raimondii* have undergone the hexaploidization event (*γ*-WGD) shared by the eudicot and a cotton-specific whole genome duplication [47]. To shed light on the mechanism about the expansion of the SOD gene family, potential duplication events involved in the evolution of* G. arboretum* and* G. raimondii* genomes were analyzed. We investigated whether traceable genome duplication events have contributed to the expansion of the SOD gene family. According to the basis of protein sequence, 4 pairs and 7 pairs of putative paralogous SOD genes were identified in* G. arboretum* and* G. raimondii,* respectively, accounting for more than 70% of the entire SOD gene family. Thereby, the result supported the hypothesis that putative gene duplication events were the main causes of the expansion of the gene family. Interestingly, we found* GrCSD4* participated in two segmental duplication events and no tandem duplication events were observed in these duplicated pairs.

Our results showed that the SOD members in the same clade of phylogenetic tree exhibited a very similar exon/intron distribution pattern. For example, the* GaMSD1* and* GrMSD1* had the same numbers of exon/intron and similar length of exon. However, our result was not consistent with the previous study that plant SODS showed the highly conserved intron pattern for most cytosol and chloroplast* SOD*s containing 7 introns [48]. By our analysis, only 4 SOD genes (GaCSD2, GaCSD4, GrCAD2, and GrCAD4) possessed 7 introns. Divergences of cotton SOD gene structures might be produced by 3 major mechanisms including exon/intron, gain/loss, and insertion/deletion [49]. The divergences of cotton SOD gene structure could be a reason of cotton unique phenotypes compared to other plants.

Previous reports demonstrated that ROS accumulation under environmental stresses could affect cellular functions by damaging nucleic acid and oxidizing proteins. Plant could balance ROS level by ROS-scavenging systems that mainly involved superoxide dismutase [50]. Our results indicate the SOD genes might play an important role for cotton on balancing the ROS accumulation and defense against abiotic stresses.

This study involved a systematic analysis of the SOD gene family in the G. arboretum and* G. raimondii* genome, including gene classification and the analysis of phylogenetic relationships, chromosomal distributions, gene expansion, gene structures, and motif compositions, as well as their expression patterns under different abiotic stresses and at different tissues in cotton. We identified 10 SOD coding genes within* G. arboreum* and* G. raimondii*. genome. Phylogenetic reconstruction indicates that SOD genes were obviously clustered into 3 subfamilies, including Cu-Zn-SOD(6), Fe-SOD(2), and Mn-SOD(2). Gene structures and conserved motifs analysis revealed that SOD gene in the same subfamily possessed similar exon/intron arrangement and composition of motifs. Segmental duplications significantly contribute to SOD gene family expansion. Finally we investigated the tissue-specific expression profiles of SOD genes in* G. arboretum*. We also found that most of SOD genes were highly expressed in leaf; it indicates that they maybe participated in the development process of cotton leaf.

Taken together, this study has imparted new insights into the putative functions of SOD gene family in cotton. Findings of the present investigation could help in understanding the role of SOD gene family in various aspects of the life cycle of cotton.

## Supplementary Material

Supplementary Figure S1. Domain analysis of SOD genes in cotton.
Supplementary Table S1. The duplicated genes of SOD gene family and their date of duplication events.
Supplementary Table S2. The sequences of the 14 motifs by motif analysis of *GaSOD* genes.
Supplementary Table S3. List of primers used in quantitative real time-PCR expression analysis.

## Figures and Tables

**Table 1:** List of primers used in quantitative real time PCR expression analysis.

Name	Forward primers (5′-3′)	Reverse primers (5′-3′)
GaCSD1	CCCTGATGGAGTTGCTGAGG	CCTGCATTCCCTGTCGTCTT
GaCSD2	CTACCGTGACTGGGAACCTTT	CACAGCCATCATCACCAACAG
GaCSD3	ACCCACCGGTTCTTTTCTCC	GTCAACGTGACAACGCCTTC
GaCSD4	ACCTGGAAAACACGGTTGGT	AAACAGCTATTGACCGCCCA
GaCSD5	CAGCCACTTCTCACATTATCTCC	AGAACTTGTGGGTCTGAAGGGTT
GaCSD6	TCAACAGGACCTCACTTCAA	ACGACCGCTCTTCCAATA
GaFSD1	CCCCTTGTTTGGGACTACTT	TCGGTTTGCCCTCTTCTT
GaFSD2	ATTGGGGTGTGCATCATCGT	TCATGGTTCCACACCTCTGC
GaMSD1	TGAGCCTCCACATGGTTCTTTG	ATCCTGATTTGCAGTGGTTTCG
GaMSD2	CACGATGCTGCTGCCTCTGTC	ACCTCCGCCGTTGAACTTGAT

**Table 2:** Characteristics of SOD genes from *G. arboretum* and *G. raimondii*. AA: amino acid; pI: the theoretical isoelectric point of proteins; Mw: the theoretical molecular weight of proteins.

Gene name	Gene symbol	Length (aa)	MW (Da)	pI	Chr. location	Subcellular location
GaCSD1	Cotton_A_30467	151	15475.4	6.82	Chr3: 58314910–58317769	Chloroplast
GaCSD2	Cotton_A_24238	152	15339.8	5.3	Chr5: 34195003–34196511	Chloroplast
GaCSD3	Cotton_A_32487	214	22104.8	6.02	Chr6: 66118430–66120830	Cytoplasm
GaCSD4	Cotton_A_09949	328	34642.9	5.68	Chr7: 15011474–15014254	Chloroplast
GaCSD5	Cotton_A_36793	224	23232.3	6.48	Chr8: 1110059–1112675	Cytoplasm
GaCSD6	Cotton_A_21978	202	20894.5	5.73	Chr8: 126578106–126579386	Cytoplasm
GaFSD1	Cotton_A_03623	309	35645.8	4.84	Chr1: 66936792–66939676	Chloroplast
GaFSD2	Cotton_A_26478	256	29328.5	6.17	Chr5: 2099667–81015489	Chloroplast
GaMSD1	Cotton_A_04050	230	25704.2	7.14	Chr6: 81012722–81015489	Mitochondria
GaMSD2	Cotton_A_21263	231	25941.7	7.23	Chr8: 3364759–3366909	Mitochondria
GrCSD1	Gorai.007G261100.1	161	16454.4	6.58	Chr7: 43021117–43024908	Cytoplasm
GrCSD2	Gorai.013G192900.6	152	15182.7	5.47	Chr13: 28906676–28910311	Cytoplasm
GrCSD3	Gorai.006G104900.2	214	222122.9	6.02	Chr6: 34720308–34723354	Chloroplast
GrCSD4	Gorai.004G205500.1	330	34793.1	6.00	Chr4: 53571645–5008855	Chloroplast
GrCSD5	Gorai.009G090300.2	181	18769.1	6.17	Chr9: 6585677–6588476	Chloroplast
GrCSD6	Gorai.013G116800.4	214	222122.9	6.02	Chr6: 28906676–28910311	Chloroplast
GrFSD1	Gorai.001G052700.1	309	35528.8	4.85	Chr1: 50041596–5008855	Chloroplast
GrFSD2	Gorai.013G068400.1	256	29256.3	5.98	Chr13: 7919077–7922556	Chloroplast
GrMSD1	Gorai.009G293300.3	230	25764.3	8.60	Chr9: 25425806–25429022	Mitochondria
GrMSD2	Gorai.011G207900.2	231	26010.7	8.81	Chr11: 49976860–49979522	Mitochondria

## Additional Points

*Highlights*. They are genome-wide analysis of SOD gene family in cotton.

## References

[1] Ruan Y.-L., Llewellyn D. J., Furbank R. T. (2003). Suppression of sucrose synthase gene expression represses cotton fiber cell initiation, elongation, and seed development. *The Plant Cell*.

[2] Wang S., Wang J.-W., Yu N. (2004). Control of plant trichome development by a cotton fiber MYB gene. *The Plant Cell*.

[3] Shi Y.-H., Zhu S.-W., Mao X.-Z. (2006). Transcriptome profiling, molecular biological, and physiological studies reveal a major role for ethylene in cotton fiber cell elongation. *The Plant Cell*.

[4] Qin Y.-M., Zhu Y.-X. (2011). How cotton fibers elongate: a tale of linear cell-growth mode. *Current Opinion in Plant Biology*.

[5] Paterson A. H., Wendel J. F., Gundlach H. (2012). Repeated polyploidization of *Gossypium* genomes and the evolution of spinnable cotton fibres. *Nature*.

[6] Soltis D. E., Soltis P. S., Tate J. A. (2004). Advances in the study of polyploidy since plant speciation. *New Phytologist*.

[7] Sunilkumar G., Campbell L. M., Puckhaber L., Stipanovic R. D., Rathore K. S. (2006). Engineering cottonseed for use in human nutrition by tissue-specific reduction of toxic gossypol. *Proceedings of the National Academy of Sciences of the United States of America*.

[8] Senchina D. S., Alvarez I., Cronn R. C. (2003). Rate variation among nuclear genes and the age of polyploidy in *Gossypium*. *Molecular Biology and Evolution*.

[9] Karuppanapandian T., Moon J.-C., Kim C., Manoharan K., Kim W. (2011). Reactive oxygen species in plants: their generation, signal transduction, and scavenging mechanisms. *Australian Journal of Crop Science*.

[10] Bhattacharjee S. (2012). The language of reactive oxygen species signaling in plants. *Journal of Botany*.

[11] Quan L.-J., Zhang B., Shi W.-W., Li H.-Y. (2008). Hydrogen peroxide in plants: a versatile molecule of the reactive oxygen species network. *Journal of Integrative Plant Biology*.

[12] Gechev T. S., Van Breusegem F., Stone J. M., Denev I., Laloi C. (2006). Reactive oxygen species as signals that modulate plant stress responses and programmed cell death. *BioEssays*.

[13] Sugimoto M., Oono Y., Gusev O. (2014). Genome-wide expression analysis of reactive oxygen species gene network in Mizuna plants grown in long-term spaceflight. *BMC Plant Biology*.

[14] Noctor G., Foyer C. H. (1998). Ascorbate and glutathione: keeping active oxygen under control. *Annual Review of Plant Physiology and Plant Molecular Biology*.

[15] Perry J. J. P., Shin D. S., Getzoff E. D., Tainer J. A. (2010). The structural biochemistry of the superoxide dismutases. *Biochimica et Biophysica Acta - Proteins and Proteomics*.

[16] Alscher R. G., Erturk N., Heath L. S. (2002). Role of superoxide dismutases (SODs) in controlling oxidative stress in plants. *Journal of Experimental Botany*.

[17] Miller A.-F. (2012). Superoxide dismutases: ancient enzymes and new insights. *FEBS Letters*.

[18] Abreu I. A., Cabelli D. E. (2010). Superoxide dismutases-a review of the metal-associated mechanistic variations. *Biochimica et Biophysica Acta—Proteins and Proteomics*.

[19] Møller I. M. (2001). Plant mitochondria and oxidative stress: electron transport, NADPH turnover, and metabolism of reactive oxygen species. *Annual Review of Plant Biology*.

[20] Luo X., Wu J., Li Y. (2013). Synergistic effects of GhSOD1 and GhCAT1 overexpression in cotton chloroplasts on enhancing tolerance to methyl viologen and salt stresses. *PLoS ONE*.

[21] Kim H. J., Kato N., Kim S., Triplett B. (2008). Cu/Zn superoxide dismutases in developing cotton fibers: evidence for an extracellular form. *Planta*.

[22] Potikha T. S., Collins C. C., Johnson D. I., Delmer D. P., Levine A. (1999). The involvement of hydrogen peroxide in the differentiation of secondary walls in cotton fibers. *Plant Physiology*.

[23] Kliebenstein D. J., Monde R.-A., Last R. L. (1998). Superoxide dismutase in *Arabidopsis*: an eclectic enzyme family with disparate regulation and protein localization. *Plant Physiology*.

[24] Filiz E., Tombuloğlu H. (2015). Genome-wide distribution of superoxide dismutase (SOD) gene families in Sorghum bicolor. *Turkish Journal of Biology*.

[25] Nath K., Kumar S., Poudyal R. S. (2014). Developmental stage-dependent differential gene expression of superoxide dismutase isoenzymes and their localization and physical interaction network in rice (*Oryza sativa* L.). *Genes & Genomics*.

[26] Wilkins M. R., Gasteiger E., Bairoch A. (1999). Protein identification and analysis tools in the ExPASy server. *Methods in Molecular Biology*.

[27] Horton P., Park K.-J., Obayashi T. (2007). WoLF PSORT: protein localization predictor. *Nucleic Acids Research*.

[28] Emanuelsson O., Nielsen H., Brunak S., von Heijne G. (2000). Predicting subcellular localization of proteins based on their N-terminal amino acid sequence. *Journal of Molecular Biology*.

[29] Liu R. H., Meng J. L. (2003). MapDraw: a microsoft excel macro for drawing genetic linkage maps based on given genetic linkage data. *Yi Chuan*.

[30] Yang S., Zhang X., Yue J.-X., Tian D., Chen J.-Q. (2008). Recent duplications dominate NBS-encoding gene expansion in two woody species. *Molecular Genetics and Genomics*.

[31] Gu Z., Cavalcanti A., Chen F.-C., Bouman P., Li W.-H. (2002). Extent of gene duplication in the genomes of *Drosophila*, nematode, and yeast. *Molecular Biology and Evolution*.

[32] Blanc G., Wolfe K. H. (2004). Widespread paleopolyploidy in model plant species inferred from age distributions of duplicate genes. *Plant Cell*.

[33] Christianson J. A., Llewellyn D. J., Dennis E. S., Wilson I. W. (2010). Global gene expression responses to waterlogging in roots and leaves of cotton (*Gossypium hirsutum* L.). *Plant and Cell Physiology*.

[34] Padmalatha K. V., Dhandapani G., Kanakachari M. (2012). Genome-wide transcriptomic analysis of cotton under drought stress reveal significant down-regulation of genes and pathways involved in fibre elongation and up-regulation of defense responsive genes. *Plant Molecular Biology*.

[35] Molina-Rueda J. J., Tsai C. J., Kirby E. G. (2013). The *Populus* superoxide dismutase gene family and its responses to drought stress in transgenic poplar overexpressing a pine cytosolic glutamine synthetase (GS1a). *PLoS ONE*.

[36] Desai A., Chee P. W., Rong J., May O. L., Paterson A. H. (2006). Chromosome structural changes in diploid and tetraploid A genomes of *Gossypium*. *Genome*.

[37] Perry J. J. P., Shin D. S., Getzoff E. D., Tainer J. A. (2010). The structural biochemistry of the superoxide dismutases. *Biochimica et Biophysica Acta (BBA)—Proteins and Proteomics*.

[38] Fan J., Chen K., Amombo E., Hu Z., Chen L., Fu J. (2015). Physiological and molecular mechanism of Nitric Oxide (NO) involved in bermudagrass response to cold stress. *PLoS ONE*.

[39] Shafi A., Chauhan R., Gill T. (2015). Expression of SOD and APX genes positively regulates secondary cell wall biosynthesis and promotes plant growth and yield in *Arabidopsis* under salt stress. *Plant Molecular Biology*.

[40] Rizhsky L., Liang H., Mittler R. (2002). The combined effect of drought stress and heat shock on gene expression in tobacco. *Plant Physiology*.

[41] Pan Y., Wu L. J., Yu Z. L. (2006). Effect of salt and drought stress on antioxidant enzymes activities and SOD isoenzymes of liquorice (*Glycyrrhiza uralensis* Fisch). *Plant Growth Regulation*.

[42] Lv S., Yang A., Zhang K., Wang L., Zhang J. (2007). Increase of glycinebetaine synthesis improves drought tolerance in cotton. *Molecular Breeding*.

[43] Foyer C. H., Shigeoka S. (2011). Understanding oxidative stress and antioxidant functions to enhance photosynthesis. *Plant Physiology*.

[44] Kim H. J., Triplett B. A. (2001). Cotton fiber growth in planta and in vitro. Models for plant cell elongation and cell wall biogenesis. *Plant Physiology*.

[45] Hu G., Koh J., Yoo M.-J., Grupp K., Chen S., Wendel J. F. (2013). Proteomic profiling of developing cotton fibers from wild and domesticated *Gossypium barbadense*. *New Phytologist*.

[46] Cannon S. B., Mitra A., Baumgarten A., Young N. D., May G. (2004). The roles of segmental and tandem gene duplication in the evolution of large gene families in *Arabidopsis thaliana*. *BMC Plant Biology*.

[47] Li F., Fan G., Wang K. (2014). Genome sequence of the cultivated cotton *Gossypium arboreum*. *Nature Genetics*.

[48] Fink R. C., Scandalios J. G. (2002). Molecular evolution and structure-function relationships of the superoxide dismutase gene families in angiosperms and their relationship to other eukaryotic and prokaryotic superoxide dismutases. *Archives of Biochemistry and Biophysics*.

[49] Xu G., Guo C., Shan H., Kong H. (2012). Divergence of duplicate genes in exon-intron structure. *Proceedings of the National Academy of Sciences of the United States of America*.

[50] Mittler R., Vanderauwera S., Gollery M., Van Breusegem F. (2004). Reactive oxygen gene network of plants. *Trends in Plant Science*.

