# Supplementary material for: Genome-Wide Characterization and Expression Profiles of the Superoxide Dismutase Gene Family in Gossypium
Source: Int J Genomics. 2016 Aug 31;2016:8740901. doi: 10.1155/2016/8740901 (PMC5021877; doi:10.1155/2016/8740901)
Supplement: Supplementary file 1 — Supplementary Figure S1. Domain analysis of SOD genes in cotton. Supplementary Table S1. The duplicated genes of SOD gene family and their date of duplication events. Supplementary Table S2. The sequences of the 14 motifs by motif analysis of GaSOD genes. Supplementary Table S3. List of primers used in quantitative real time-PCR expression analysis. [file 8740901.f1.pdf]

**Supplemental figure and table legends**

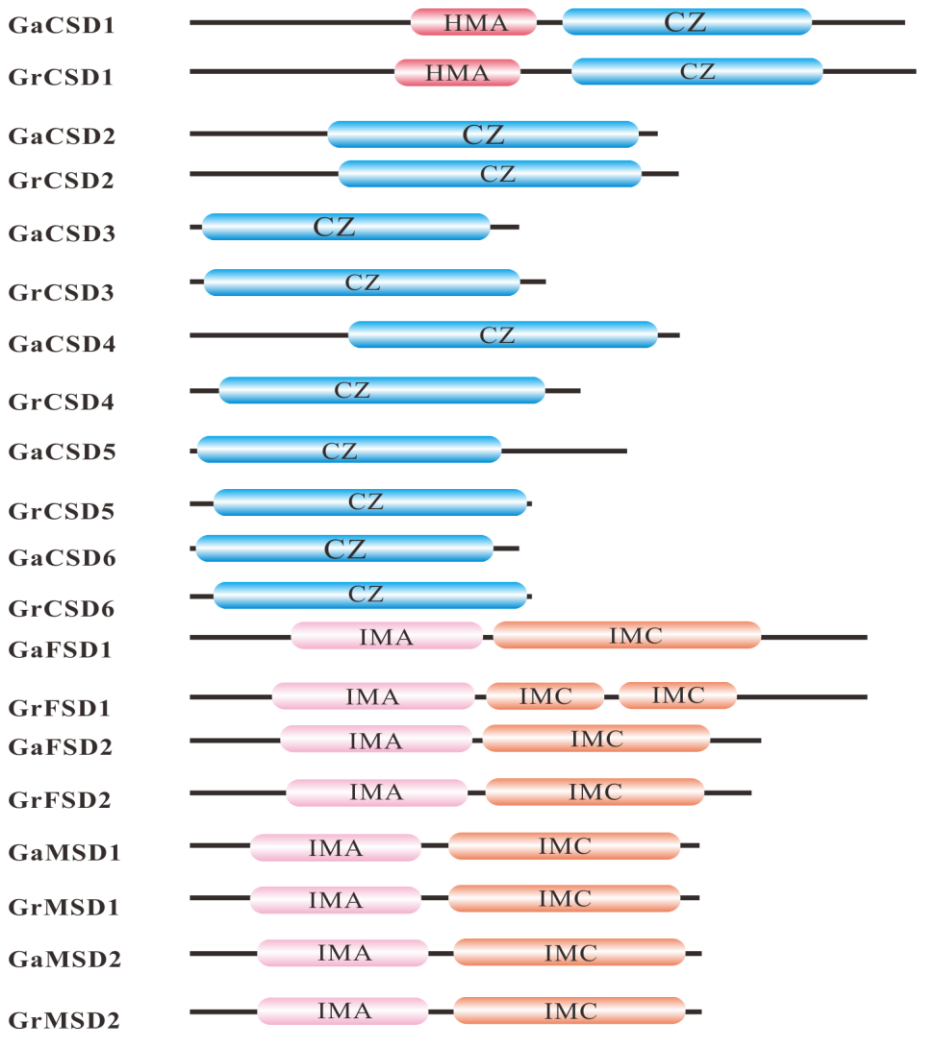


**Supplementary Figure S1** Domain analysis of SOD genes in cotton

**Supplementary Table S1.** The duplicated genes of SOD gene family and their date of duplication events

| Gene pairs | | ka | ks | Ka/ks | T= Ks/2λ |
|---|---|---|---|---|---|
| GaCSD2 | GaCSD6 | 0.039 | 0.5586 | 0.07 | 18.62Mya |
| GaCSD3 | GaCSD5 | 0.0975 | 0.3729 | 0.26 | 12.43Mya |
| GaFSD1 | GaFSD2 | 0.4156 | 0.9221 | 0.45 | 30.73Mya |
| GaMSD1 | GaMSD2 | 0.0429 | 0.4071 | 0.11 | 13.57Mya |
| GrCSD2 | GrCSD6 | 0.0328 | 0.4939 | 0.07 | 16.46Mya |
| GrCSD3 | GrCSD4 | 0.6042 | 0.5853 | 1.03 | 19.51Mya |
| GrCSD3 | GrCSD5 | 0.0662 | 0.3630 | 0.18 | 12.1Mya |
| GrCSD1 | GrCSD6 | 0.4787 | 0.2739 | 1.75 | 9.13Mya |
| GrFSD1 | GrFSD2 | 0.4136 | 0.9845 | 0.42 | 32.827Mya |
| GrMSD1 | GrMSD2 | 0.0529 | 0.4198 | 0.13 | 13.99Mya |

**Supplementary Table S2.** The sequences of the 14 motifs by motif analysis of *GaSOD* genes.

| Motif No. | Length (aa) | E-value | Multilevel Consensus Sequence | No. of SOD proteins in which motif is present | SOD proteins in which motif is present |
|---|---|---|---|---|---|
| Motif 1 | 91 | 5.4E-514 | [PA]HFNP[ANL][GKNR][KM][ETD]HGAP[ES]D[EGV][NVE]RHAGDLGN[IV][ITV][AV][GN][ADP]DG[VCS]A[ES][AFV][ST]I[VKT]D[KNW]QIPL[ST]G[PQ][NH][SA][IV][ILV]GRA[VF]VVH[AE][DL][PE]DDLGKGGHELS[KL][TS]TGNAG[GA]R[VL][AG]CG[IV][IV]GL[QT] | 10 | GaCSD1,GaCSD2, GaCSD3,GaCSD5 GaCSD6,GrCSD1, GrCSD2,GrCSD3 GrCSD5,GrCSD6 |
| Motif 2 | 42 | 1.00E-156 | [AQ][TY][FY][SEG]L[KP][DAT][LP]PY[DNP][LY][GDN][AD]LEP[AHY][IM]S[GKR][ER][IAT][LM][QE][LVY]H[HW][GQ][KV]HH[QR][TD][YF][IV][TDE]N[LY]NK[AQ] | 8 | GaFSD1,GaFSD2, GaMSD1,GaMSD2 GrFSD1,GrFSD2, GrMSD1,GrMSD2 |
| Motif 3 | 41 | 1.40E-213 | [LF]TQEGDGPTTV[NT][GV][NR]I[ST]GL[KTS]PGLHGFH[LVI]H[EA][LY]GDTTNGCMSTG | 11 | GaCSD2,GaCSD3, GaCSD4,GaCSD5 GaCSD6,GrCSD1, GrCSD2,GrCSD3 GrCSD4,GrCSD5, GrCSD6 |
| Motif 4 | 47 | 2.20E-131 | GG[EDG][PKM]P[HKNS][GAL][SDG]L[GL][WDE][ALQ]I[DE][TR][DHN]FGS[FL][ET][SNR][FL][IR][EQ][KE][FM][NKV][AE][AE][AG][AL][AST][LQ][FQ]GSGW[VA]WL[GAV][LVY][KDN][KAR][ENQ] | 8 | GaFSD1,GaFSD2, GaMSD1,GaMSD2 GrFSD1,GrFSD2, GrMSD1,GrMSD2 |
| Motif 5 | 31 | 6.00E-123 | [PT][IKLN][GV][PW][DHN][LDY][VFI]P[LI]L[GNT][IL]D[VM]WEHAYYL[DQ][YF][KQ]N[VD][RK][PA][DR]Y[LI] | | GaFSD1,GaFSD2, GaMSD1,GaMSD2 GrFSD1,GrFSD2, GrMSD1,GrMSD2 |
| Motif 6 | 41 | 1.00E-72 | QLH[DE]AIQKGDSSTVVKLQSAIKFNGGGH[IV]NHSIFWKNLAPI | 4 | GaMSD1,GaMSD2. GrMSD1,GrMSD2 |
| Motif 7 | 29 | 5.20E-51 | TYN[KN]G[DN][IP]LP[AE]FNNAA[EQ][AV]WNHDFFWESM[KQ]P | 4 | GaFSD1,GaFSD2, GrFSD1,GrFSD2 |
| Motif 8 | 41 | 1.30E-46 | L[PS][RN][QP][SV][LV][SR][IL][AL][AG][TN][IS]P[KV]K[QT][FM][SN][VE][AFL][AL][VE][QST][KT][KG][AR][VK]A[VR]L[KI]G[DNQ][SG][EKV][VP]E[GD][VF][VL][TV] | | GaCSD3,GaCSD4, GaCSD5,GrCSD3 GrCSD4,GrCSD5 |
| Motif 9 | 21 | 2.80E-40 | [KV][NF][IMV][WE]K[VL][IV][NS]W[KE][YT][AV][SN][ET][VR][YL]EK[EA][CKS]A | 6 | GaFSD1,GrFSD1, GaMSD1,GaMSD2 GrMSD1,GrMSD2 |
| Motif 10 | 80 | 1.40E-27 | TLDVDKNGE[AS]FYTGV[EK]QQLRVADLIGRSIAVYETEDRSDPGLTAAVIARSAGVGENYKKICACDGTTIWEASDKDFVT[ST]K | 2 | GaCSD4,GrCSD4 |
| Motif 11 | | 3.10E-27 | RNSSHFPNAKSLPFLSSPPNRLGL[IT]RNFATSP[IT]ALNMDSPS[ST][HN]HNPSQENGSLPDLLTEYMVDMKCEGCVTAVKNKLQTV | 2 | GaCSD4,GrCSD4 |
| Motif 12 | 21 | 4.80E-23 | [MA]A[LT][RT]S[LMS][VA]T[RS][KLR]T[LF]T[LI][AGP][LS][NL][SG]TRL | 6 | GaFSD1,GrFSD1, GaMSD1,GaMSD2 GrMSD1,GrMSD2 |
| Motif 13 | 41 | 5.20E-16 | RAAEREMEEERRRKEEEEGKPTNEE[DE]DDDDLEMYVDNDNDD | 2 | GaFSD1,GrFSD1 |
| Motif 14 | 15 | 2.50E-15 | K[AKP][LV][VA][VL][LE][ST][ST]F[EN][GQ][VD][SP][LG][TV] | 11 | GaCSD2,GaCSD3, GaCSD5,GaCSD6 GrCSD2,GrCSD3, GrCSD6,GaMSD1 GaMSD2,GrMSD1, GrMSD2 |

**Supplementary Table S3.** List of primers used in quantitative real time-PCR expression analysis.

| Name | Forward primers (5′- 3′) | Reverse primers (5′- 3′) |
|---|---|---|
| GaCSD1 | CCCTGATGGAGTTGCTGAGG | CCTGCATTCCCTGTCGTCTT |
| GaCSD2 | CTACCGTGACTGGGAACCTTT | CACAGCCATCATCACCAACAG |
| GaCSD3 | ACCCACCGGTTCTTTTCTCC | GTCAACGTGACAACGCCTTC |
| GaCSD4 | ACCTGGAAAACACGGTTGGT | AAACAGCTATTGACCGCCCA |
| GaCSD5 | CAGCCACTTCTCACATTATCTCC | AGAACTTGTGGGTCTGAAGGGTT |
| GaCSD6 | TCAACAGGACCTCACTTCAA | ACGACCGCTCTTCCAATA |
| GaFSD1 | CCCCTTGTTTGGGACTACTT | TCGGTTTGCCCTCTTCTT |
| GaFSD2 | ATTGGGGTGTGCATCATCGT | TCATGGTTCCACACCTCTGC |
| GaMSD1 | TGAGCCTCCACATGGTTCTTTG | ATCCTGATTTGCAGTGGTTTCG |
| GaMSD2 | CACGATGCTGCTGCCTCTGTC | ACCTCCGCCGTTGAACTTGAT |
